# Tissue-specific mitochondrial heteroplasmy at position 16,093 within the same individual

**DOI:** 10.1007/s00294-013-0398-6

**Published:** 2013-07-11

**Authors:** Kaarel Krjutškov, Marina Koltšina, Kelli Grand, Urmo Võsa, Martin Sauk, Neeme Tõnisson, Andres Salumets

**Affiliations:** 1 Competence Centre on Reproductive Medicine and Biology, Tartu, Estonia; 2 Estonian Genome Center, University of Tartu, Tartu, Estonia; 3 Department of Biosciences and Nutrition, Karolinska Institute, Huddinge, Sweden; 4 Department of Biotechnology, University of Tartu, Tartu, Estonia; 5 Department of Genetics, United Laboratories, Tartu University Hospital, Tartu, Estonia; 6 Department of Obstetrics and Gynaecology, University of Tartu, Tartu, Estonia; 7 Institute of Bio- and Translational Medicine, University of Tartu, Tartu, Estonia

**Keywords:** Human tissue-specific mtDNA heteroplasmy, Illumina sequencing

## Abstract

Human mitochondrial DNA (mtDNA) research has entered a massively parallel sequencing (MPS) era, providing deep insight into mtDNA genomics and molecular diagnostics. Analysis can simultaneously include coding and control regions, many samples can be studied in parallel, and even minor heteroplasmic changes can be detected. We investigated heteroplasmy using 16 different tissues from three unrelated males aged 40–54 years at the time of death. mtDNA was enriched using two independent overlapping long-range PCR amplicons and analysed by employing illumina paired-end sequencing. Point mutation heteroplasmy at position 16,093 (m.16093T > C) in the non-coding regulatory region showed great variability among one of the studied individuals; heteroplasmy extended from 5.1 % in red bone marrow to 62.0 % in the bladder. Red (5.1 %) and yellow bone marrow (8.9 %) clustered into one group and two arteries and two aortas from different locations into another (31.2–50.9 %), giving an ontogenetic explanation for the formation of somatic mitochondrial heteroplasmy. Our results demonstrate that multi-tissue screening using MPS provides surprising data even when there is a limited number (3) of study subjects and they give reason to speculate that mtDNA heteroplasmic frequency, distribution, and even its possible role in complex diseases or phenotypes seem to be underestimated.

**Electronic supplementary material:**

The online version of this article (doi:10.1007/s00294-013-0398-6) contains supplementary material, which is available to authorized users.

## Background

Mitochondria that host 37 genes within 16.6 kb of their genome (mtDNA) are the power engines of the cell. Mitochondrial DNA (mtDNA) gene regulation and replication start from the non-coding control region, D-loop, that is 1,124 bp long from positions 16,024–576. The D-loop has three hypervariable regions (HVRI-III) and contains 25 % of the variable sites, even though it represents only 7 % of the total mtDNA length (Sosa et al. 2012). Mitochondrial diseases associated with mtDNA mutations and its phenotypic heterogeneity can be explained by mtDNA heteroplasmy—the mixture of more than one type of mtDNA at a cellular, tissue or organism level. The 100-fold higher mutation rate of mtDNA than nuclear DNA is caused by a combination of oxidative microenvironment, lack of histones, and rudimentary repair mechanisms, leading to mtDNA mutation accumulation during aging. Although mtDNA is maternally inherited in mammals, heteroplasmy levels vary markedly between a mother and each of her offspring. This means that asymptomatic mothers with medium to low heteroplasmy levels can have an affected child with much higher levels of the same heteroplasmic mutation (>30 %), which may cause a mitochondrial disease (Zhang et al. 2012).

Massively parallel sequencing (MPS) allows simultaneous analysis of the complete mtDNA and provides annotation and mutant load estimation of each position. Taking into account the variable sequencing depth of published data, small sample sizes, read mapping and data filtering criteria, and heteroplasmy detection thresholds, there is no consensus of opinion as regards average heteroplasmic frequency distribution among individuals and in populations. Full-length mtDNA analysis from 131 individuals identified 40 point heteroplasmies and indels at 10 % minor allele frequency (MAF) or higher in blood or saliva, making 0.3 heteroplasmies per individual (Li et al. 2010). In contrast, 1.8 heteroplasmies per individual at >10 % MAF were identified using 40 immortalized lymphoblastoid cell line samples (Sosa et al. 2012). In recent reports (Andrew et al. 2011; Goto et al. 2011; Avital et al. 2012), variable heteroplasmy in the human body has been demonstrated to exist within a limited number of tissues such as blood, skeletal muscle and buccal epithelium, leaving the rest of the organism out of focus. As multiple tissue analysis in healthy individuals is highly complicated, data on whole body mtDNA heteroplasmy is still far from clear.

In this study, we were able to investigate three unrelated males by employing MPS technology to detect mtDNA heteroplasmy using 16 different tissues taken during post-mortem examination. We show a great range of heteroplasmy variance at position 16,093, where the lowest values were detected in red and yellow bone marrow tissue and the highest in the bladder. The data contribute to mtDNA genetics at human body level and explain the possible formation and expansion of heteroplasmy during post-zygotic embryonic development.

## Methods

The Research Ethics Committee of the University of Tartu approved the collection of tissue samples for research. The written informed consent was obtained from next-of-kin to post-mortem individuals to collect the tissue panel during the autopsy. Encoded body panel was collected from three males of 40 (sample KT538), 53 (KA522) and 54 (SJ600) years of age at the time of death. Donors KT538 and KA522 had died of acute cardiovascular insufficiency due to myocardial infarction and SJ600 of cerebellar haemorrhage. The tissue samples were collected within 8 h of post-mortem and consisted of abdominal adipose tissue, lienal artery, bone, yellow bone marrow, red bone marrow, coronary artery, gastric mucosa, joint cartilage, thoracic lymph node, tonsils, bladder, gall bladder, abdominal aorta, thoracic aorta, medulla oblongata and nervus ischiadicus (Table 1). DNA extraction was carried out according to the recommendations of the NucleoSpin Tissue DNA extraction kit manufacturer (Macherey-Nagel, Düren, Germany), with DNA concentrations and quality parameters provided in Supplementary Table 1. Four anonymous genomic DNA samples for replication sequencing were provided by the Estonian Genome Center of the University of Tartu (EGC). Whole mtDNA was amplified in two amplicons (8,225 and 9,447 bp) by PCR using two pairs of primers. PCR amplicons were enzymatically fragmented, prepared as a 48-plex sequencing library and analysed using an Illumina HiSeq2000 instrument with 101 bp paired-end reads according to the manufacturer’s protocols. Confirmation of mtDNA position 16,093 heteroplasmic samples and the 300–320 D-loop region in three individuals was performed by Sanger sequencing. The data were analysed using freely available software. All details of methods used are provided in Supplementary Methods.

**Table 1:** Heteroplasmy overview at position 16,093 of individual SJ600

Tissue	Sequencing depth	Major allele	C^a^	Minor allele	T^a^	MAF (%)
Bladder	12,339	C	4,691	T^b^	7,645	62.0
Lienal artery	5,343	C	2,625	T^b^	2,717	50.9
Coronary artery	17,265	C	11,123	T^b^	6,139	35.6
Thoracic aorta	6,870	C	4,446	T^b^	2,422	35.3
Abdominal aorta	9,828	C	6,762	T^b^	3,061	31.2
Nervus ischiadicus	8,391	C	6,190	T^b^	2,200	26.2
Gall bladder	5,433	C	4,041	T^b^	1,391	25.6
Bone	11,603	C	9,047	T^b^	2,555	22.0
Gastric mucosa	11,954	C	9,692	T^b^	2,222	18.6
Abdominal adipose tissue	3,724	C	3,039	T^b^	685	18.4
Medulla oblongata	12,985	C	11,266	T^b^	1,717	13.2
Thoracic lymph node	14,600	C	12,672	T^b^	1,926	13.2
Tonsils	10,827	C	9,752	T^b^	1,073	9.9
Joint cartilage	14,444	C	13,074	T^b^	1,368	9.5
Yellow bone marrow	18,223	C	16,607	T^b^	1,613	8.9
Red bone marrow	11,650	C	11,051	T^b^	598	5.1

^a^Allele read count

^b^The reference allele according to the revised Cambridge reference sequence (rCRS)

## Results

**Fig. 1:**
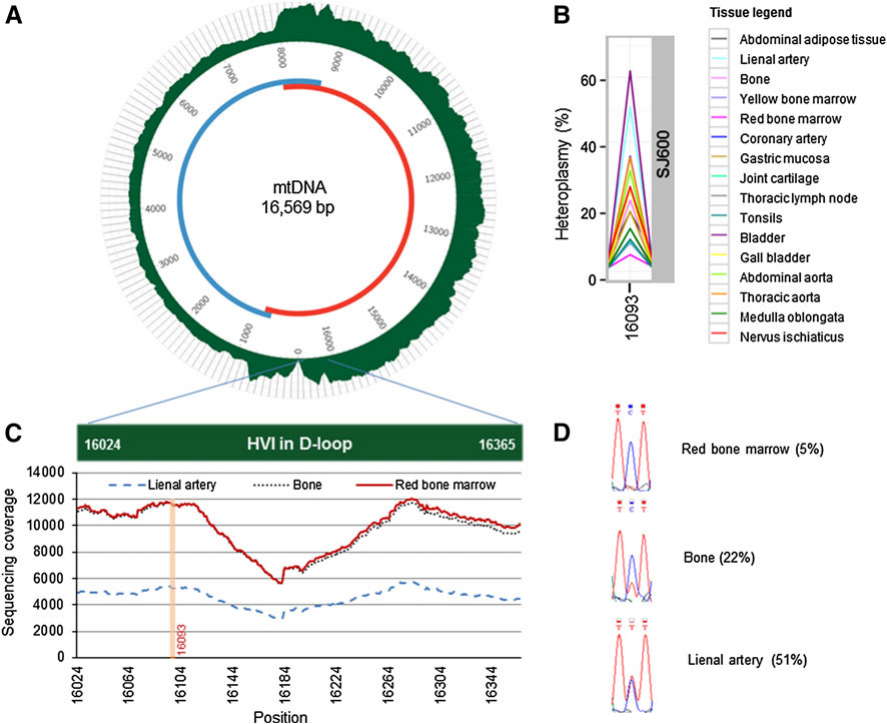
Full mtDNA amplification and the confirmation of results. **a** mtDNA was amplified using two overlapping PCR products, *blue* and *red circles*. Average coverage density is shown (*green*). **b** Position 16,093 heteroplasmy over the SJ600 body panel. **c** Sequencing coverage in hypervariable region (HVRI) of D-loop. Coverage in the three different tissues of individual SJ600 is shown. **d** Sanger sequencing confirmation of position 16,093 heteroplasmy. The percentage is from MPS analysis and chromatogram from Sanger sequencing

A total of 260 million paired-end reads were generated for 16-tissue body panels in three unrelated males (48 samples in total) and for four replicates (8 samples). Each sample library preparation was started from 0.5 ± 0.1 μg of PCR product, but demultiplexing revealed 22-fold differences between sample raw reads (Supplementary Fig. 1). Mapping and read trimming increased it to 49-fold, but quality score-based filtering and PCR duplicate removal finally reduced sample variability to 25-fold. The post-filtering mean quality score was 33.5 and 84 % of reads (median) had a quality score of ≥30. 80.2 % (median) of reads mapped to mtDNA reference and 41.5 % of reads (median) were removed afterwards during the PCR duplicate filtering step. The average MAF fraction over the full mtDNA sequence is <0.15 % (Supplementary Table 2). PCR amplification generated unequal coverage over mtDNA, preferring one amplicon to another and bringing out two overlapping regions in amplicons (Fig. 1a). In KA522, the joint cartilage tissue sample was removed as a result of low-sequencing coverage and quality, caused by lower (*p* = 0.0003) purity of the original DNA sample (Supplementary Table 1).

Forty-seven samples were mapped against a reference sequence to investigate heteroplasmy in mtDNA. Preliminary analysis revealed heteroplasmy with MAF of <10 % at mtDNA positions 302, 309, 310, and 316 in the three studied DNA body panels but Sanger resequencing of poly-C tracks in mtDNA bases 302–310 and 312–317 showed no heteroplasmy in this region, and confirmed only KT538 C insertion at mtDNA position 309 (named C309CC, Supplementary Fig. 2). That demonstrates that MPS mapping output needs Sanger confirmation under these conditions. The significant heteroplasmy (>10 %) was detected only in individual SJ600 at D-loop position 16,093 (m.16093T > C) (Fig. 1b). Red and yellow bone marrow had 5.1 and 8.9 % heteroplasmy, respectively. Abdominal aorta, thoracic aorta, coronary artery and lienal artery clustered as one group and showed heteroplasmy in the range of 31.2–50.9 %, while 62.0 % mtDNA heteroplasmy was detected in the bladder (Table 1). Three tissues with variable heteroplasmy of 5–50 % were examined in detail and confirmed by independent Sanger sequencing (Fig. 1c–d). Two other individuals (KT538 and KA522) had no heteroplasmy at position 16,093.

Four blood DNAs from the EGC were analysed as duplicates to show the assay reproducibility. The median sequencing depth of all eight samples varied from 4,832 to 11,531 and at least 99.5 % of all the nucleotides had >1,000 fold sequencing depth (Supplementary Table 3). In total, eight heteroplasmic positions were detected at >5 % MAF. Heteroplasmic MAF variance between the replicates was 1.5–25.8 % and the correlation coefficients were 0.88–0.99 (Supplementary Fig. 3).

## Discussion

mtDNA is highly represented in somatic cells, making it as a considerable DNA source after nuclear DNA. Nevertheless, the “total length” of mtDNA per cell is up to 20 Mb, which is still 400 times less than the amount of diploid nuclear DNA and therefore mtDNA needs specific enrichment before the MPS analysis. Despite the success of capture-probe-based mtDNA enrichment (Li et al. 2012), PCR is still the “gold standard” for selection and amplification of small, targeted DNA regions, like mtDNA. In addition, the results indicate that PCR-introduced errors should not be a significant problem as regards this approach (Li et al. 2010; Zaragoza et al. 2010). We used 22 PCR cycles to reduce possible bias accumulation and ensured an efficient PCR yield with 0.2–1.2 μg of input DNA. The size-selected library was repaired and amplified using ten additional cycles, which were too intense because 41.5 % of mapped reads were eliminated during PCR duplicate filtering (Supplementary Fig. 1).

The heteroplasmy threshold is an important parameter for accurate detection of heteroplasmy. The threshold calculations based on nuclear DNA control libraries demonstrated that the average proportion of mutations per base was 0.06 % and no base was mutated at greater than 0.8 % frequency (He et al. 2010). A similarly low threshold (1.3 %) was reported using synthetic DNA fragments with variable allelic ratios (Zhang et al. 2012), providing the acceptable 5–10 % cut-off for heteroplasmy (Li et al. 2010; Sosa et al. 2012). At the same time, attention should be paid to the fact that artificial quality control DNAs are only useful for assessing indexed library amplification, cluster generation and MPS itself, but are not suitable for sample enrichment and DNA quality issues. In our study, the detected variability at position 16,093 was confirmed by Sanger sequencing at 5, 22 and 51 % MPS-detected heteroplasmy levels. As expected, 5 % heteroplasmy is not detectable by dideoxy-terminator sequencing, 22 % heteroplasmy remains unquantified, and 51 % heteroplasmy correlates as a 1:1 polymorphic allelic ratio (Fig. 1d). As our experiment was designed without internal controls to detect the MPS sensitivity threshold, we are not able to report the number of true-positive heteroplasmies without a confirmation experiment. Nevertheless, we detected a great range of position 16,093 heteroplasmy in the human body, contributing to a previous multi-tissue analysis (He et al. 2010). In that previous study, somatic heteroplasmy ranging 7–91 % was found in one of the two study patients. However, the authors studied significantly fewer tissue samples from the same autopsy individual, with little overlap to the panel used in our study. Furthermore, colorectal cancer as the cause of death in (He et al. 2010), could influence the extent of somatic heteroplasmy.

Unfortunately our analysed tissues did not include blood samples, but according to other recent reports reflecting low-level heteroplasmy in blood (Andrew et al. 2011; Avital et al. 2012; Payne et al. 2012), we can speculate that blood (leukocytes) may have lower heteroplasmy level than the rest of human body. Because red bone marrow consists mainly of hematopoietic tissue giving rise to all blood cell types, we used red bone marrow (heteroplasmy 5.1 %) as a possible alternative to blood in our study. Also, it is suggested that rapidly dividing tissues such as blood cells or bone marrow may have the ability to eliminate cells with mtDNA mutations during life, whereas “post-mitotic” tissues that no longer divide do not eliminate these cells and may instead favour accumulation of mutant mtDNA genomes. Hence, this could clarify why some inherited mtDNA mutations can be lost from the blood but be detected in other post-mitotic tissues (Payne et al. 2012). Our findings are in line with this, demonstrating very low frequency of heteroplasmy in rapidly dividing red and yellow bone marrow tissues, with 5 and 9 % of heteroplasmy, respectively.

According to our findings, position 16,093 was heteroplasmic in all 16 studied tissues in one individual, varying from 5.1 % in red bone marrow to 62.0 % in the bladder. The differences between organ groups can be explained by random segregation of mtDNA during oogenesis and in somatic tissue development. It is now believed that somatic segregation may lead to high levels of mutant mtDNA in some progenitor cells and low levels in others. In addition, due to the high copy number of mtDNAs, a mutation in some of the mtDNA results in a mixture of variant mitochondrial genomes within a mitochondrion, cell, tissue, organ, or individual. However, it is impossible to predict the cell types or tissues to which mutant molecules will segregate during the ontogenesis. It is likely that such heteroplasmic variability in our study is a result of random genetic drift and not strongly associated with certain developmental stages or tissue differentiation processes. These findings could explain the unequal distribution of the different genotypes, not only between the three germinal layers (endoderm, mesoderm and ectoderm), but also between derivatives of the same germinal layer. In our panel that mostly contains tissues of mesodermal origin, heteroplasmy at position 16,093 differed by more than 40 % between two mesodermal derivatives—bone marrow and lienal artery. In contrast, the same heteroplasmic frequency (9 %) was present in tissues of different origin, such as tonsils and joint cartilage, which are of endodermal and mesodermal origin, respectively. Interestingly, our results clearly demonstrated similar high-heteroplasmic frequency in various blood vessels—abdominal aorta, thoracic aorta, coronary artery and lienal artery, ranging 31–51 %. Arteries carry oxygenated blood away from the heart to the tissues that require oxygen. These findings may partially be interpreted by the production of oxygen radicals during oxidative phosphorylation and common mesodermal origin. The fact that in bladder tissue we observed the highest frequency of heteroplasmy could be explained by its tissue-specific prevalence, as also indicated by the high incidence of mtDNA mutations in bladder cancer (Guney et al. 2012).

In conclusion, our results demonstrate that screening of a tissue panel using MPS technology provides surprising data that reveals somatic heteroplasmy even among the limited number of study subjects. Our findings give clear reason to speculate that mtDNA heteroplasmic frequency, distribution, and even its possible role in complex diseases or phenotypes could be severely underestimated, evoking the need for more thorough tissue-based screenings at different age groups to provide more comprehensive understanding about its biological and clinical importance.

## Electronic supplementary material

Below is the link to the electronic supplementary material.

Supplementary material 1 (PDF 1742 kb)
