# Supplementary material for: Tissue-specific mitochondrial heteroplasmy at position 16,093 within the same individual
Source: Curr Genet. 2013 Jul 11;60(1):11–6. doi: 10.1007/s00294-013-0398-6 (PMC3895442; doi:10.1007/s00294-013-0398-6)
Supplement: Supplementary file 1 — Supplementary material 1 (PDF 1742 kb) [file 294_2013_398_MOESM1_ESM.pdf]

## Supplementary methods

### Study subjects

The study samples were collected by a pathologist at the North Estonia Medical Centre in 2004. The anonymously encoded body panel was collected from 3 males of 40 (sample KT538), 53 (KA522) and 54 years of age (SJ600) at the time of death. Donors KT538 and KA522 had died of acute cardiovascular insufficiency due to myocardial infarction, and SJ600 of cerebellar haemorrhage. The tissue samples were collected within 8 hours post-mortem and consisted of abdominal adipose tissue, lienal artery, bone, yellow bone marrow, red bone marrow, coronary artery, gastric mucosa, joint cartilage, thoracic lymph node, tonsils, bladder, gall bladder, abdominal aorta, thoracic aorta, *medulla oblongata* and *nervus ischiaticus* (Table 1), and were stored at -80°C. The Research Ethics Committee of the University of Tartu approved the collection of tissue samples for research. Written informed consent was obtained from next-of-kin to post-mortem individuals in order to collect the tissue panel during the autopsy.

### DNA extraction, mtDNA amplification and sequencing

DNA extraction was carried out according to the recommendations of the NucleoSpin™ Tissue DNA extraction kit manufacturer (Macherey-Nagel, Düren, Germany), with minor modifications. Human tissue (25 mg) was cut into small pieces, 180 µl Buffer T1 and 25 µl Proteinase K (all reagents from Macherey-Nagel) were added and the tissue was incubated for 20 hours at 56°C. After brief vortexing, 200 µl Buffer B3 was added and the tissue was incubated for 10 min at 70°C. Ethanol (210 µl, 96%) was added and the sample was centrifuged for 1 min at 13,000×*g*. The sample was washed first with 500 µl Buffer BW and centrifuged for 1 min at 13,000×*g*. The second wash was performed by adding 600 µl Buffer B5 and centrifuged for 1 min at 13,000×*g*. The DNA was then dried with centrifugation for 1 min at 13,000×*g*. DNA was eluted in 50 µl pre-warmed (70°C) Buffer BE. The elution step was repeated once. Sample concentrations, and 260/280 nm and 260/230 nm ratios were measured using an ND-1000 spectrophotometer (Nano-Drop, Wilmington, USA) and the data are summarised in Supplementary Table 1. Four anonymous genomic DNA samples for technical replication sequencing were provided by the Estonian Genome Center of the University of Tartu. DNA was extracted from venous blood using a standard protocol. The studied samples were determined to have 140–180 ng/µl concentration in ND-1000 spectrophotometry (Nano-Drop) before PCR. Whole mtDNA was amplified in two amplicons (8,225 and 9,447 bp) by PCR using two pairs of primers.

PCR amplicons were enzymatically fragmented, prepared as a 48-plex sequencing library and analysed using an Illumina HiSeq2000 instrument with 101 bp paired-end reads according to the manufacturer's protocols (Illumina Inc., USA).

***DNA extraction from venous blood***

To select out leucocytes for DNA extraction, 30 ml of non-frozen fresh blood was centrifuged at 1,000 rpm (Eppendorf centrifuge 5810R in all following steps) for 6 min at RT. The upper plasma was discarded. Sample was placed to 50 ml centrifuge tube, added Solvent 1 (155 mM $NH_4Cl$, 10 mM $KHCO_3$ (pH 8.0), 1 mM EDTA-$Na_2$) to fill up 50 ml level, mixed and incubated on ice for 10 min. After centrifugation at 1,800 rpm for 10 min at RT, solvent was discarded, leaving up to 1 cm liquid level. 25 ml of Solvent 1 was added. Sample was mixed and centrifuged at 1,600 rpm for 10 min at RT. Solvent was discarded, leaving approximately 0.5 cm liquid in the tube. For homogeneous suspension, 2 ml of Solvent 1 was added and mixed to dissolve the leucocytes from the bottom of the tube. To separate the genomic DNA from leucocytes, 20 ml of Solvent 2 (10 mM Tris-Cl (pH 8.0), 25 mM EDTA, 2% SDS) was added, mixed to homogenous suspension and incubated at 38ºC for 20 min. Sample was cooled to 20ºC. 8.5 ml 10 M ammonium acetate was added to sample, mixed and centrifuged at 3,500 rpm for 10 min at RT. Solvent was then discarded to new clean 50 ml tubes with 20 ml isopropanol and mixed gently for 5 min. DNA strings were separated and purified with 70% ethanol for 5 min. 6 ml of TE buffer (10 mM Tris-Cl (pH 7.5), 1 mM EDTA) was added to dried sample and incubated 30 min at 56ºC on gentle vortex. DNA stayed on vortex for a week or with higher concentrations until the solution was complete at RT.

***MtDNA amplification***

The whole mtDNA was amplified in two amplicons, 8,225 and 9,447 bp by PCR using two pairs of primers. The following primers were used: CTCCTCAAAGCAATACACTG and TGGGTGGTTGGTGTAAATGA (amplicon one) and ACGAGTACACCGACTACGGC and AGGCTAAGCGTTTTGAGCTG (amplicon two) (these and all the following oligos were ordered from Metabion (Martinsried, Germany)). The primer sequences used were verified against the entire human genome using Blast, in order to have no nuclear genome amplification. The amplification reactions were checked by agarose gel electrophoresis. Two overlapping regions of both amplicons covered 1,103 bp. The PCR products were amplified separately in 11 µl single-plex reactions using 1× Long PCR buffer with 1.5 $MgCl_2$ (Thermo Fisher Scientific, MA, US), 0.18 mM dNTPs, 3.6% DMSO, 1 U Long PCR Enzyme Mix (Thermo), 0.9 µM forward and reverse primers of amplicon one and two, respectively, and 0.2–1.2 µg of genomic DNA.

The amplifications were carried out using GeneAmp PCR System 9700 (Applied Biosystems, Foster City, CA, USA) according to the following 22 cycle protocol: initial denaturation at 94°C/3 min; 10 cycles of 94°C/20 sec, 52°C/30 sec, 68°C/10 min, and 12 cycles of 94°C/20 sec, 50°C/30 sec, 68°C/11 min, and final extension 10 min at 68°C. Amplicons one and two of each DNA sample were mixed and cleaned-up by NucleoSpin Extract II kit (Macherey-Nagel) with minor modifications: 100 µl Binding Buffer was added to 20 µl PCR mixture and finally eluted with 50 µl pre-warmed (80°C) elution buffer. All purified samples were quantified by ND-1000 spectrophotometer (NanoDrop) and balanced to 0.5±0.1 µg in 50 µl for further library preparation.

***Sample preparation and sequencing***

PCR amplicons were enzymatically fragmented using NEBNext dsDNA Fragmentase (New England Biolabs, MA, US). The reactions were carried out in 58 µl volume using 50 µl (0.5 µg) balanced sample, 6 µl 10× Fragmentase Reaction Buffer, 1 µl (100 mg/ml) BSA, 1 µl dsDNA Fragmentase enzyme mixture, and incubated after a brief vortexing for 2 h at 37°C. The fragmentation reactions were stopped by adding 120 µl NucleoSpin Extract II Binding buffer (Macherey-Nagel) and purified according to the manufacturer's protocol. The products were finally eluted in 14 µl Elution Buffer (Macherey-Nagel). End repair and other library preparation manipulations were performed with NEXTflex PCR-free DNA Sequencing Kit (Bioo Scientific, Austin, TX, US) after multiple modifications, where the reaction volumes were reduced three times to save on the library preparation costs. The end repair was performed in 16.7 µl using 14 µl of fragmented DNA, 2.7 µl End Repair Mix (Bioo Scientific), and incubated after a brief vortexing for 30 min at RT. Reactions were stopped by adding 100 µl NucleoSpin Extract II Binding buffer and purified according to manufacturer's protocol. The products were finally eluted in 10 µl Elution Buffer. For fragment 3' A-tailing, 1 µl of Adenylation Mix (Bioo Scientific) was added to the previously purified products and reactions incubated after a brief vortexing for 30 min at 37°C. Illumina-compatible NEXTflex PCR-free 48-plex barcode oligonucleotide adapters (Bioo Scientific) were ligated. For that, 0.7 µl of adapters and 11 µl Ligation Mix (Bioo Scientific) were added to A-tailed products. Incubation was carried out for 15 min at 22°C and the ligase was inactivated by incubating for 10 min at 70°C. All the samples were mixed and purified by Agencourt AMPure XP beads (Beckman Coulter, Brea, CA, US). All bead-based purification steps were performed at RT. Briefly, equal amount of beads were added to the library pool, mixed and incubated for 10 min. The tubes were placed to magnetic rack for 20 min and pipetted carefully for the maximum magnetic effect. The clear supernatant (approximately 90% of volume) was removed and 1 ml of freshly prepared 80% ethanol was added. Ethanol was removed after 30 sec and the washing step was repeated once. After the full drying of ethanol, beads were eluted in 100 µl Elution buffer and incubated for 5 min out of magnetic rack. The magnetic rack was placed to collect beads for 5 min and the clear supernatant was transferred to a clean tube for further purification. The second bead-based purification was performed with smaller volumes of reagents: 100 µl of beads, 300 µl of ethanol and 20 µl of Elution buffer.

The samples were size-selected on a 2% agarose gel, recovering the 200–600 bp range by the NucleoSpin Extract II gel extraction protocol. The heating step was replaced by a 15 min vigorous agitation and samples were eluted in 15 µl.

The library was PCR amplified for concentration and repairment before the sequencing cluster generation in 50 µl volume using 25 µl 2× Phusion Mastermix (Thermo), 0.5 µM primers (AATGATACGGCGACCACCGAGATCTACAC and CAAGCAGAAGACGGCATACGAGAT), and 5 µl of size-selected library. The amplification was carried out with Piko Thermal Cycler (Thermo) with initial denaturation at 98°C/30 sec; 10 cycles of 98°C/15 sec, 65°C/20 sec, and final extension 30 sec at 65°C. AMPure beads were used for the final purification as described previously: 50 µl beads, 300 µl ethanol and 20 µl Elution buffer. Ready 48-plex libraries were visualised by Agilent 2100 Bioanalyzer (Agilent Technologies, Santa Clara,

CA, US) and quantified by KAPA DNA Quantification Standards and Primer Premix Kit (Kapa Biosystems, Cape Town, South Africa). The cluster formation and sequencing-by-synthesis were performed using Illumina HiSeq2000 instrument, 101 bp paired-end reads, and V3 reagents according to the manufacturer's protocols (Illumina, Inc.).

**Dye terminator confirmation sequencing**

Confirmation of mtDNA position 16,093 heteroplasmic samples and the 300–320 D-loop region in three individuals was performed by Sanger sequencing using ACACCAGTCTTGTAAACCGG and AACGTGTGGGCTATTTAGGC primers for mtDNA position 16,093, and CATTTGGTATTTCGTCTGGG and AGGGTGAACTCACTGGAACG for the 300–320 region. Sanger dye terminator sequencing with the Applied Biosystems 3130xl DNA Analyzer following the recommended instructions was used. Sequencing primer CTCCACCATTAGCACCCAAAG for position 16,093, and ATTGAATGTCTGCACAGCCACTTTCCAC for the 300–320 region were used. The sequencing data was analysed using the CromasPro Version 1.41 software package.

**Mapping, quantification and heteroplasmy calling**

The pooled samples were demultiplexed using CASAVA 1.8 software (Illumina) allowing zero mismatches within six barcode nucleotides. Raw reads were mapped to the revised Cambridge reference (rCRS) sequence NC_012920 using the BWA (0.6.0) algorithm (Li and Durbin, 2009), discarding reads with quality scores less than 30. SAM-tools (0.1.18) (Li et al., 2009) was used to generate SAM and BAM datasets and to remove PCR duplicates. Heteroplasmy was detected using MitoBamAnnotator software (Zhidkov et al,. 2011) with default options and the data was visualised using an R software package with minimum 1,000-fold coverage threshold. An Interactive Genomics Viewer (version2.1, 10 GB) (Robinson et al., 2011) was used for visualisation of filtered reads.

***References***

Li H, Durbin R (2009) Fast and accurate short read alignment with Burrows-Wheeler transform. Bioinformatics 25: 1754-1760.

Li H, Handsaker B, Wysoker A, Fennell T, Ruan J, et al. (2009) The Sequence Alignment/Map format and SAMtools. Bioinformatics 25: 2078-2079.

Robinson JT, Thorvaldsdottir H, Winckler W, Guttman M, Lander ES, et al. (2011) Integrative genomics viewer. Nat Biotechnol 29: 24-26.

Zhidkov I, Nagar T, Mishmar D, Rubin E (2011) MitoBamAnnotator: A web-based tool for detecting and annotating heteroplasmy in human mitochondrial DNA sequences. Mitochondrion 11: 924-928.

**Supplementary Figure 1.** Read distribution through different massively-parallel sequencing filtering steps; raw reads (yellow), reads after quality score filtering (light green), and PCR duplicate removal (green).

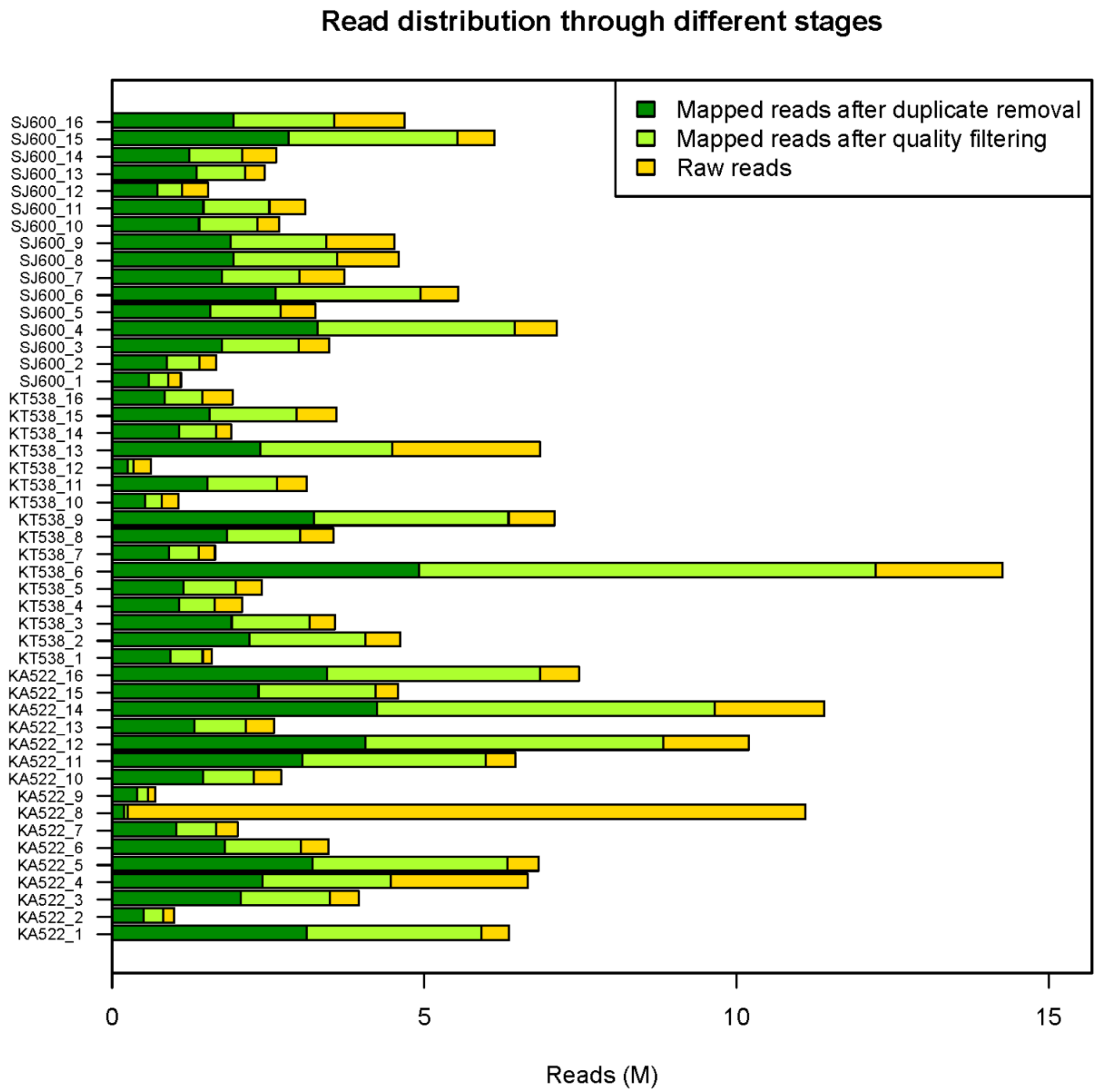

**Supplementary Figure 2.** Sanger re-sequencing of region 300-320 bp. Poly-C tracks from 304-310 and 312-317 caused a read mapping issue and the region seems highly heterogeneous after preliminary analysis. Sanger sequencing revealed homoplasmic poly-C track in DNA KA522 and SJ600, and position 309 C-CC insertion (C309CC) in KT538.

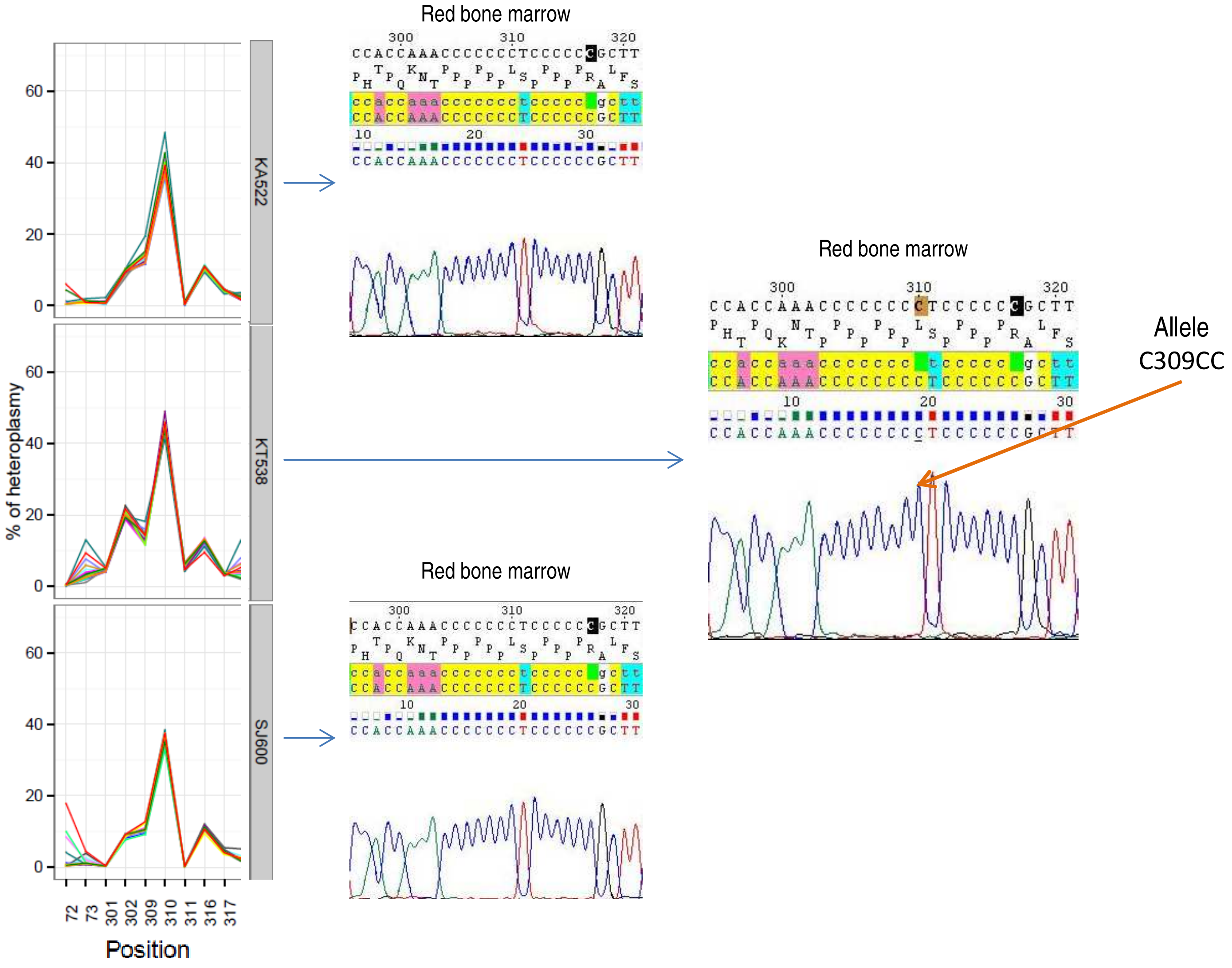

**Supplementary Figure 3.** Four replicates and correlation coefficients (R). Four samples were prepared and analyzed in parallel. After data analysis the heteroplasmy MAF values of both replicates (Replication A and B) were plotted and correlation coefficient was calculated.

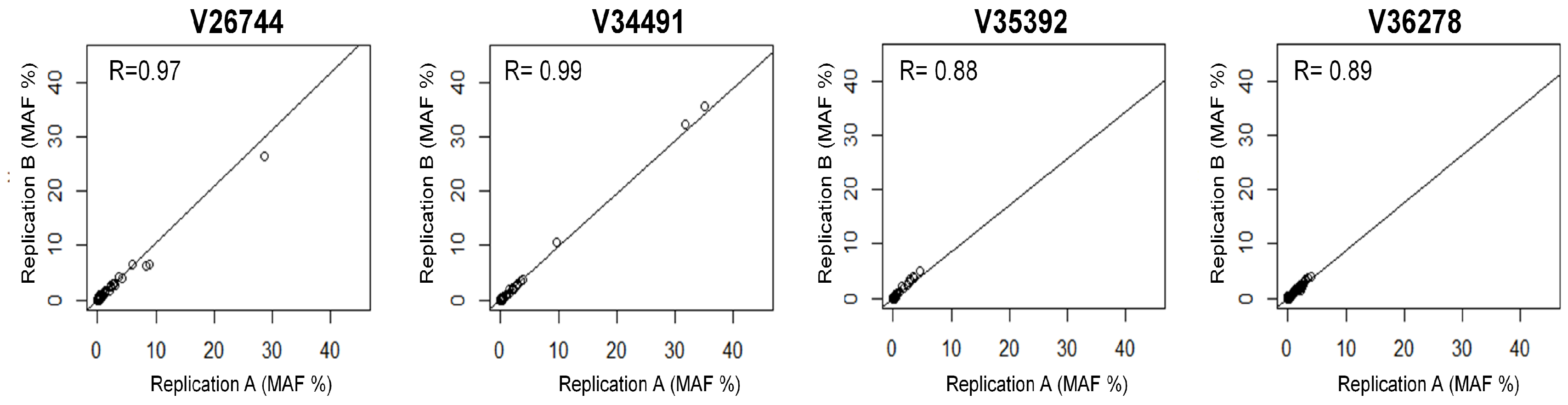

**Supplementary Table 1.** Names and numeration of studied tissues. Studied DNAs, ages, DNA concentrations and quality parameters using 260/280 and 260/230 nm ratios.

| | | KT538, 40 years | | | KA522, 53 years | | | SJ600, 54 years | | |
|---|---|---|---|---|---|---|---|---|---|---|
| **Tissue** | **Numeration** | **ng/µl** | **260/280** | **260/230** | **ng/µl** | **260/280** | **260/230** | **ng/µl** | **260/280** | **260/230** |
| Abdominal adipose tissue | 1 | 52 | 1.74 | 1.65 | 73 | 1.86 | 1.81 | 54 | 1.87 | 2.07 |
| Lienal artery | 2 | 58 | 1.75 | 2.16 | 54 | 1.83 | 1.85 | 70 | 1.78 | 2.08 |
| Bone | 3 | 67 | 1.97 | 2.19 | 70 | 1.59 | 1.37 | 75 | 1.88 | 1.98 |
| Yellow bone marrow | 4 | 271 | 1.86 | 2.36 | 64 | 1.66 | 1.15 | 69 | 1.84 | 2.15 |
| Red bone marrow | 5 | 76 | 1.79 | 2.03 | 192 | 1.86 | 2.28 | 145 | 1.82 | 2.3 |
| Coronary artery | 6 | 65 | 1.74 | 1.13 | 72 | 1.83 | 2.06 | 79 | 1.87 | 1.93 |
| Gastric mucosa | 7 | 55 | 1.84 | 2.17 | 81 | 1.78 | 2.1 | 228 | 1.85 | 2.38 |
| Joint cartilage | 8 | 90 | 1.81 | 1.93 | 54 | **1.37** | 1.38 | 73 | 1.75 | 1.59 |
| Thoracic lymph node | 9 | 91 | 1.86 | 2.36 | 98 | 1.84 | 2.04 | 220 | 1.84 | 2.36 |
| Tonsils | 10 | 95 | 1.8 | 2.1 | 285 | 1.89 | 2.37 | 87 | 1.8 | 2.21 |
| Bladder | 11 | 62 | 1.82 | 2.08 | 79 | 1.84 | 2.07 | 80 | 1.73 | 1.76 |
| Gall bladder | 12 | 82 | 1.88 | 2.25 | 97 | 1.85 | 2.25 | 62 | 1.76 | 2.23 |
| Abdominal aorta | 13 | 52 | 1.74 | 1.45 | 58 | 1.77 | 1.6 | 63 | 1.8 | 1.52 |
| Thoracic aorta | 14 | 52 | 1.76 | 1.84 | 65 | 1.62 | 1.43 | 51 | 1.56 | 2.25 |
| *Medulla oblongata* | 15 | 70 | 1.55 | 1.53 | 114 | 1.55 | 1.26 | 74 | 1.76 | 1.58 |
| *Nervus ischiaticus* | 16 | 36 | 1.7 | 1.25 | 67 | 1.88 | 2.1 | 59 | 1.75 | 1.93 |

**Supplementary Table 2**. Average coverage and median of minor allele frequency (MAF) of three studied individuals.

| | **Average coverage** | **Fraction below 1000× (%)** | **Average/median MAF fraction** (%)** |
|---|---|---|---|
| KT538 | 7119 | 8.15 | 0.14/0.09 |
| KA522* | 10033 | 1.03 | 0.13/0.09 |
| SJ600 | 7745 | 2.64 | 0.14/0.10 |

* one tissue sample was excluded from the analysis

** calculated in regions >1000× coverage

**Supplementary Table 3.** Main characterisations of four technical replicates and unconfirmed heteroplasmies MAF ≥5% **(A).** Unconfirmed variances ≥1% of both replicates of all four samples are listed in detail in part (**B).**

**A**

| DNA | Total reads (M) | Mapping (%) | Median depth | >1000× coverage (%) | Heteroplasmies MAF ≥5% | | | |
|---|---|---|---|---|---|---|---|---|
| | | | | | *16,189* | *16,188* | *16,19* | *16,182* |
| 26744_A | 1.85 | 60.7 | 4831 | 99.5 | 26.45 | 6.58 | 6.53 | 6.22 |
| 26744_B | 5.94 | 43.7 | 11454 | 99.8 | 28.67 | 8.87 | 6.06 | 8.33 |
| | | | | | *16,093* | *16,497* | *146* | |
| 34491_A | 3.7 | 52.7 | 8699 | 99.8 | 35.6 | 32.39 | 10.63 | |
| 34491_B | 5.16 | 50.2 | 11531 | 99.8 | 35.1 | 31.66 | 9.66 | |
| | | | | | *14,833* | | | |
| 35392_A | 4.79 | 47 | 9935 | 99.8 | 5.03 | | | |
| 35392_B | 3.34 | 56.6 | 7973 | 99.7 | 4.61 | | | |
| | | | | | - | | | |
| 36278_A | 2.22 | 57.1 | 5462 | 99.6 | - | | | |
| 36278_B | 4.69 | 48.5 | 9466 | 99.8 | - | | | |

**B**

**26744_A**

| pos | ref | total | MAF (%) |
|---|---|---|---|
| 16189 | T | 3723 | 26.45 |
| 16188 | C | 3726 | 6.58 |
| 16190 | C | 3667 | 6.53 |
| 16182 | A | 3691 | 6.22 |
| 10306 | A | 3814 | 4.32 |
| 3109 | T | 5736 | 3.03 |
| 6419 | A | 4287 | 2.97 |
| 2487 | A | 4496 | 2.96 |
| 16194 | A | 3657 | 2.72 |
| 3492 | A | 3178 | 2.58 |
| 3110 | C | 5743 | 2.46 |
| 318 | T | 1657 | 1.75 |
| 16195 | T | 3649 | 1.62 |
| 3003 | A | 4828 | 1.4 |
| 3105 | A | 5632 | 1.21 |

**26744_B**

| pos | ref | total | MAF (%) |
|---|---|---|---|
| 16189 | T | 8603 | 28.67 |
| 16188 | C | 8610 | 8.87 |
| 16182 | A | 8483 | 8.33 |
| 16190 | C | 8511 | 6.06 |
| 10306 | A | 8974 | 3.56 |
| 3492 | A | 8148 | 3.05 |
| 3109 | T | 13923 | 2.87 |
| 6419 | A | 10533 | 2.82 |
| 2487 | A | 10897 | 2.73 |
| 3110 | C | 13950 | 2.33 |
| 16194 | A | 8514 | 2.32 |
| 318 | T | 4353 | 1.99 |
| 3003 | A | 12364 | 1.35 |
| 16195 | T | 8511 | 1.24 |
| 3105 | A | 13722 | 1.21 |

**34491_A**

| pos | ref | total | MAF (%) |
|---|---|---|---|
| 16093 | T | 9236 | 35.6 |
| 16497 | A | 3386 | 32.39 |
| 146 | T | 3975 | 10.63 |
| 10306 | A | 7045 | 3.46 |
| 3492 | A | 5729 | 3.02 |
| 2487 | A | 7868 | 2.8 |
| 6419 | A | 7353 | 2.57 |
| 3109 | T | 9852 | 2.46 |
| 16304 | T | 5616 | 2.19 |
| 3110 | C | 9882 | 2.05 |
| 3243 | A | 9353 | 2 |
| 318 | T | 3379 | 1.91 |
| 3105 | A | 9700 | 1.21 |

**34491_B**

| pos | ref | total | MAF (%) |
|---|---|---|---|
| 16093 | T | 11727 | 35.1 |
| 16497 | A | 4383 | 31.66 |
| 146 | T | 5090 | 9.66 |
| 10306 | A | 9184 | 3.48 |
| 3492 | A | 7996 | 2.9 |
| 6419 | A | 10273 | 2.83 |
| 2487 | A | 10424 | 2.8 |
| 3109 | T | 13791 | 2.57 |
| 3243 | A | 12927 | 2.17 |
| 16304 | T | 6912 | 2.07 |
| 3110 | C | 13840 | 2.04 |
| 318 | T | 4574 | 1.53 |
| 3105 | A | 13589 | 1.44 |

| 35392_A | | | |
|---|---|---|---|
| pos | ref | total | MAF (%) |
| 14833 | A | 9560 | 5.03 |
| 10306 | A | 7881 | 3.92 |
| 3492 | A | 6862 | 3.45 |
| 2487 | A | 9540 | 3.1 |
| 6419 | A | 8923 | 2.9 |
| 3109 | T | 12065 | 2.51 |
| 318 | T | 3805 | 2.09 |
| 3110 | C | 12101 | 1.98 |
| 3105 | A | 11840 | 1.27 |

| 35392_B | | | |
|---|---|---|---|
| pos | ref | total | MAF (%) |
| 14833 | A | 7258 | 4.61 |
| 10306 | A | 5967 | 3.48 |
| 2487 | A | 8675 | 2.97 |
| 3492 | A | 6139 | 2.87 |
| 6419 | A | 7977 | 2.64 |
| 3109 | T | 11202 | 2.49 |
| 3110 | C | 11226 | 1.89 |
| 318 | T | 2992 | 1.52 |
| 3105 | A | 11026 | 1.18 |

| 36278_A | | | |
|---|---|---|---|
| pos | ref | total | MAF (%) |
| 10306 | A | 4263 | 3.65 |
| 2487 | A | 5656 | 3.44 |
| 3492 | A | 3766 | 3.17 |
| 3109 | T | 6991 | 2.92 |
| 6419 | A | 4827 | 2.89 |
| 1888 | G | 6841 | 2.86 |
| 6663 | A | 6483 | 2.47 |
| 5147 | G | 6196 | 2.45 |
| 4216 | T | 5640 | 2.37 |
| 3110 | C | 7016 | 2.34 |
| 930 | G | 5464 | 2.33 |
| 4917 | A | 6335 | 2.19 |
| 6379 | T | 4599 | 2.12 |
| 16296 | C | 3481 | 1.96 |
| 1189 | T | 4394 | 1.9 |
| 709 | G | 5977 | 1.83 |
| 1811 | A | 7131 | 1.61 |
| 14769 | A | 5272 | 1.61 |
| 11295 | T | 5464 | 1.59 |
| 16304 | T | 3417 | 1.52 |
| 8697 | G | 9228 | 1.48 |
| 318 | T | 1982 | 1.43 |
| 3434 | A | 4465 | 1.39 |
| 3480 | A | 3158 | 1.37 |
| 16294 | C | 3517 | 1.37 |
| 3105 | A | 6862 | 1.24 |
| 16126 | T | 5327 | 1.24 |
| 14766 | C | 5349 | 1.23 |
| 14905 | G | 4675 | 1.23 |
| 7028 | C | 7003 | 1.19 |
| 11719 | G | 5251 | 1.19 |
| 15928 | G | 5758 | 1.18 |
| 8271 | A | 9754 | 1.13 |
| 5047 | T | 6973 | 1.1 |
| 15236 | A | 5450 | 1.1 |
| 10463 | T | 5842 | 1.06 |
| 11251 | A | 5327 | 1.01 |

| 36278_B | | | |
|---|---|---|---|
| pos | ref | total | MAF (%) |
| 10306 | A | 7047 | 3.4 |
| 3492 | A | 7675 | 3.02 |
| 2487 | A | 10779 | 2.97 |
| 6419 | A | 10213 | 2.8 |
| 1888 | G | 13922 | 2.77 |
| 3109 | T | 13582 | 2.64 |
| 930 | G | 11469 | 2.57 |
| 6379 | T | 10530 | 2.49 |
| 16296 | C | 5488 | 2.46 |
| 4216 | T | 11792 | 2.42 |
| 6663 | A | 13254 | 2.41 |
| 16304 | T | 5356 | 2.19 |
| 5147 | G | 12237 | 2.16 |
| 4917 | A | 13316 | 2.15 |
| 1981 | G | 13105 | 2.08 |
| 3110 | C | 13599 | 2.01 |
| 16294 | C | 5544 | 1.78 |
| 1189 | T | 9570 | 1.77 |
| 14769 | A | 7884 | 1.76 |
| 8697 | G | 15008 | 1.71 |
| 318 | T | 3403 | 1.64 |
| 1811 | A | 14451 | 1.54 |
| 3480 | A | 6512 | 1.52 |
| 3434 | A | 9021 | 1.39 |
| 7028 | C | 13879 | 1.36 |
| 11295 | T | 8726 | 1.35 |
| 3105 | A | 13365 | 1.34 |
| 15236 | A | 9125 | 1.31 |
| 14766 | C | 8019 | 1.28 |
| 15928 | G | 9672 | 1.2 |
| 10463 | T | 9640 | 1.15 |
| 14233 | A | 9162 | 1.13 |
| 8271 | A | 17552 | 1.11 |
| 13368 | G | 8503 | 1.11 |
| 11719 | G | 8415 | 1.09 |
| 5047 | T | 13336 | 1.08 |
| 15452 | C | 8576 | 1.07 |

| | | | |
|---|---|---|---|
| 13368 | G | 5091 | 1.01 |
| 15607 | A | 5356 | 1.01 |
| 16162 | A | 5242 | 1 |

| | | | |
|---|---|---|---|
| 11251 | A | 8441 | 1.06 |
| 16126 | T | 8887 | 1.06 |
| 2706 | A | 8296 | 1.05 |
| 3010 | G | 12205 | 1 |
| 6827 | T | 12148 | 1 |
